# A multi-stakeholder participatory pilot study of the data demands of the future Ethiopian dairy sector

**DOI:** 10.12688/gatesopenres.13594.3

**Published:** 2022-08-02

**Authors:** Brian D. Perry, Yacob Aklilu Gebreyes, Solomon Hailemariam, Getachew Legese, Karen Smyth, Andrew R. Peters, Fiona K. Allan, Azage Tegegne

**Affiliations:** 1 College of Medicine and Veterinary Medicine, University of Edinburgh, Edinburgh, UK; 2 Nuffield College of Clinical Medicine, University of Oxford, Oxford, UK; 3 Private consultant, 8621 Ayat Village, Addis Ababa, Ethiopia; 4 Livestock Trade Services, Kenya, Ethiopia; 5 Private Consultant, Ayat Village, Zone 1, Street 14, 1-25-8 Lemy Kura, Addis Ababa, Ethiopia; 6 International Livestock Research Institute, Addis Ababa, Ethiopia; 7 Supporting Evidence Based Interventions-Livestock, University of Edinburgh, Edinburgh, UK

**Keywords:** Ethiopia, dairy sector, stakeholders, data needs, development

## Abstract

**Background**:  This paper describes a pilot study undertaken in 2018, to determine the key data needs of each of the different Ethiopian dairy sector stakeholder groups.  The study aimed to characterise the emerging trends of dairy product production, processing, retailing and consumption in Ethiopia, and to identify and characterise current and future data needs of different stakeholders.

**Methods**:  The study undertook a mapping of the interactions between different stakeholders in the dairy sector, and an interactive evaluation of the institutional data repository and access options.  Focus group discussions and interviews were held in three regions of the country prior to a two-day workshop in the capital Addis Ababa.  Data needs were characterised by type, availability, format, level of detail, methods of dissemination, uptake and use, and the institutional arrangement, including the different roles of public and private sectors in decision making processes.

**Results**:  The study highlighted the main data needs and identified several broader institutional issues constraining the further development of the Ethiopian dairy sector.  The stakeholder groups endorsed the reactivation of a national dairy board, independent of government but closely incorporating government, and with the buy-in and membership of private sector enterprises, including producers, processers, service providers and consumers, to provide clearer facilitative leadership on the dairy industry.

**Conclusions**:  The study workshop provided a timely discussion between diverse stakeholders, including government, and several potential organisations were suggested to host and manage a national dairy database. Importantly, the reactivation of a national dairy board was strongly endorsed.  It was recommended that stakeholder links be established, sector-specific data needs be elevated to higher detail, and a national roll out of herd-specific data recording schemes was called for, to allow for effective evidence-based policies and decision making.

## Introduction

Ethiopia has a human population of around 115 million ( World Bank, 2020), and as such there is the huge potential market opportunity for milk and milk products ( Tegegne, 2018). The demand for milk and other dairy products is growing with population growth and urbanisation ( PRECISE, 2018)( Minten *et al.*, 2020), which has been regarded as a major investment opportunity (such as the Netherlands African Business Council (NABC)) ( Zijlstra *et al.*, 2015). The country has the largest livestock population in Africa, with a cattle population of more than 65 million ( Central Statistical Agency (CSA), 2020), however, it is recognised that there are several constraints to the further development of the dairy sector, including the very small population of cross bred dairy cows (estimated to be around 1.2 million, less than 2% of the cattle population)( Central Statistical Agency (CSA), 2020), the low levels of milk production, the high price of livestock feed, weak links between producers and processors, lack of a dairy board with a vision for the dairy sector, high cost of milk to the consumer, and weak links between the different stakeholders ( Yilma *et al*., 2011). These authors concluded that the major challenges were poor infrastructure network, inadequate provision of veterinary services and lack of continuous supply of animal feeds throughout the year ( Yilma *et al*., 2011). Guadu & Abebaw (2016) described the different production systems involved in dairying, and reviewed the challenges, opportunities and prospects for the sector. Similarly, the sector has been reviewed by Mihret *et al*. (2017), and Ndambi *et al*. (2018) examined geographical aspects of the sustainability of milk production in different regions of the country. These studies concluded that the major challenges included cattle health, lack of infrastructure, environmental issues, lack of access to credit, reproductive disorders and inadequate trained personnel, all of which contribute to the poor performance of dairy cattle in Ethiopia.

Milk is produced by 11.4 million milking cows that are kept within five different dairy systems: pastoral (traditional livestock farming); agro-pastoral (traditional lowland mixed livestock farming); mixed crop livestock (traditional highland mixed farming); urban and peri-urban (emerging smallholder dairy farming); and commercial (specialised commercial intensive dairy farming) ( Zijlstra *et al.*, 2015). Milk consumption is very low per capita at 19 litres per year (compared to 80 litres per year in Kenya and 160 litres per year in Sudan) ( CDAIS, 2017). The low milk production and consumption are driven by a number of factors. In rural areas the animals used by smallholder farmers are local breeds which are not selected for milk production. Animals are managed in a traditional way, depending on natural pasture and crop bi-products with no supplementary feeds; the quantity of milk produced is low. Milk in the rural setting is mainly used for household consumption and is not widely marketed, with any surplus usually converted into butter and sold in local markets ( Tegegne, 2018). The situation is different in the urban and peri-urban areas where farmers use cross-bred and exotic dairy animals, and where they have access to artificial insemination (AI), use more intensive systems, have access to commercial sources of feeds and animal health services ( Tegegne, 2018). But these farmers account for only 1% of the dairy cattle population in the country. They supply milk to consumers in major urban centres, both through the collection and distribution system of dairy processors which delivers processed products to consumers, and the raw milk traders collect and distribute raw milk mainly through informal markets. The country produces around four billion litres of milk per year ( Tegegne, 2018).

There are a number of challenges facing further development of Ethiopia’s dairy sector. The first is that local breeds provide about 1.5 litres of milk per day per cow. These cattle also have a short lactation length of about 150 days ( Tegegne, 2018). Cross bred cattle, in comparison, produce 10–15 litres of milk per day and have a lactation period of around 300 days ( Zijlstra *et al.*, 2015). Although there is a National Artificial Insemination Centre, smallholder famers have limited access to improved dairy genetics ( Ahmed *et al*., 2004)( Tegegne *et al.*, 2012) and the AI delivery system is also weak and inadequate ( Dekeba *et al.*, 2006). There are very few improved dairy animals and they are often too expensive for smallholders to purchase ( Yilma *et al.*, 2011). Feed and water are in critically short supply and livestock are mostly fed on grass hay and crop residues, most of which are poor quality ( Beyu, 2016)( Ahmed *et al.*, 2004). Supplementary feeds, such as cereal bran and oil cakes, are either too expensive or in short supply ( GebreMichael *et al.*, 2019). Dairy production requires good quality water and the availability and reliability of water is a major constraint in Ethiopia. Seasonal fluctuations in supply during the dry season, and demand (there are fasting days each week and fasting seasons by the Coptic Christian community) all affect the liquid milk chain and marketing opportunities ( Yilma *et al.*, 2011).

Despite these challenges, there are some strong and effective players operating in Ethiopia, trying to address the problems. Notably, Smart Development Works (SNV) supported dairy development initiatives ( SNV, 2018), Global Affairs Canada (GAC) supported the Livestock and Irrigation Value Chains for Ethiopian Smallholders (LIVES) project ( ILRI, 2018), the Bill and Melinda Gates Foundation (BMGF) supported the African Dairy Genetics Gain Programme (ADGG) ( ILRI communications, 2017), and the Public Private Partnership for Artificial Insemination Delivery (PAID) programme of Land O’Lakes ( Land O’Lakes, 2022). The role and position of these and other players is strengthened by the development of the Livestock Master Plan for Ethiopia ( Shapiro *et al.*, 2015), which brings potential cohesion to these differing initiatives, and a US $170 million World Bank development programme for livestock and fisheries ( World Bank, 2018). Importantly, there is also the potential for dairying in pastoral areas, which are excluded from the current World Bank funding and also from the Livestock Master Plan. A new World Bank programme of some US $350 million is under development for the pastoral areas of the country ( World Bank, 2019).

The livestock sector is viewed as a critical component of Ethiopia’s agricultural development, which is framed under the country’s Growth and Transformation Plan II (GTP II) ( National Planning Commission, 2016). Ethiopia’s Livestock Master Plan (LMP) set out predicted growth opportunities, and within the LMP an ambitious programme was drawn up for the dairy sector ( Shapiro *et al.*, 2015). Within the sector, the plan included the following targets:

•   Projected 93% increase in national cow milk production, a surplus of 2,501 million litres over projected domestic consumption requirements. This would make it possible to meet the milk production targets in the GTP II phase, exceeding the growing domestic demand for milk by 47%. The surplus could then be substituted for imported milk products and used domestically for new or additional industrial uses, or exported as milk powder or ultra-high temperature (UHT) to raise foreign exchange earnings

•   GDP contribution from dairy will increase from Ethiopian Birr (ETB) 1.1 billion to 10.0 billion

•   Adopting dairy farmers – around 1.3 million will be trained by 2020

•   There will be an increase in cross-bred cattle, from 0.9 million to 5 million

•   Synchronisation and AI interventions are proposed to raise Internal Rates of Return (IRR) values from 23.7 to 32.5%

•   Adoption of artificial insemination (AI) and synchronisation is planned to reach 32% of the reproductive female cattle by 2020

•   20,000 public and private AI technicians will be trained

•   Milk production is predicted to increase from 167 million to 1,490 million litres

In order to gain a better understanding of the feasibility of the targets, as well as monitoring and evaluation of progress in achieving the targets, there is the requirement for data. The study objective was to identify and characterise the different data needs to support effective decision making in the Ethiopian dairy sector, by building on a previous broader study ‘Landscape of Organisational Mandates and Analysis of Existing Livestock Databases in Ethiopia’ ( PRECISE, 2018), supported by the Bill and Melinda Gates Foundation (BMGF). By establishing the data needs of stakeholders, priorities and recommendations could be made for how data can be generated and managed that would best support dairy sector development.

## Methods

### Ethics and consent

No animals were involved in this work. Initially, on conducting the study, it was felt that due to the data collection being of a relatively impersonal nature, ethical approval was not necessary. The type of data participants were expected to provide related to the performance of different components of the dairy sector in Ethiopia, and no health or otherwise sensitive personal information was collected. However, on further consideration, full details and supporting documentation of the study were submitted for retrospective ethical review on 21 ^st^ February 2022 by the Human Ethical Review Committee, University of Edinburgh, the outcome of which stated that had the study been reviewed prior to commencement, ethical approval would likely have been given.

### Study design

The purposive study commenced in April 2018, involving three visits to Ethiopia (9–15 May; 28 May-2 June; 5–11 August 2018) by the first author. A literature review was conducted, followed by stakeholder focus group discussions and key informant interviews, all of which helped to inform the structure of a stakeholder workshop, which was the culmination of the study. A total of 45 participants were involved.

The researchers present for the stakeholder discussions and interviews were Brian Perry and Yacob Aklilu, and for the workshop also included Getachew Legese, Solomon Hailemariam and Azage Tegegne, all Ethiopian, male scientists with long standing experience of the Ethiopian livestock sector. Brian Perry designed and facilitated the workshop, with long-standing experience of Ethiopia and its livestock sector (he led the Independent Evaluation or the Programmes of the FAO in Ethiopia in 2010; Perry *et al*., 2011). Whilst all researchers were known professionally to the stakeholders, and therefore had built up trust, they were not known personally, with regards to bias.

### Literature review

An extensive review of the literature was conducted, to assess past and current research and development in the Ethiopian dairy sector, however there is very little available literature on the performance of the dairy sector. Sources were multiple; initially Google scholar and PubMed electronic databases were used to search for Ethiopian dairy milk production constraints which duly led on to reports and articles, and also documents were made available to the team in Ethiopia, including non-published governmental and other grey literature reports. Findings were collated in a Microsoft Word folder. Relevant literature included research articles, government documents, including the LMP, GTP-I and II and their reports, dairy sector plans and other documents to understand development efforts and plans in the sector. 

### Stakeholder scoping and mapping

Stakeholder mapping and cluster identification was carried out in three regions of Ethiopia: Addis Ababa (and its surrounds); Tigray, and Southern Nations, Nationalities, and Peoples’ Region (SNNPR). This geographical spread was undertaken in order to ensure as comprehensive engagement as possible, to pick out the key stakeholder clusters involved in dairying. Clusters were identified according to key roles undertaken by the various actors with dairy value chains in Ethiopia. Within each stakeholder cluster - government, research, feeds, genetics, health, processing, marketing, and producers - key informants were identified. Contact details were provided by the stakeholders in each of the clusters, complying with data protection legislation and ethical approval. The individuals and/or organisations operating in each cluster were identified through networking as well as the local authors provided lists of suitable participants, based on their local knowledge. Whilst there was the potential for bias, this was addressed by ensuring the widest range of participants possible.

### Checklist

After consulting previous research and better understanding the sector and major stakeholders, a checklist was developed, for use in key informant interviews and focus group discussions in order to capture key issues in line with the study objectives. The checklist was developed using past research, and was supplemented extensively from discussions with the different stakeholder representatives. Checklists provided reminders of basic issues and questions, and were amplified depending on responses obtained during interviews and discussions. The checklist is included in Extended Data.

### Consents and approval

Securing the consensus of relevant government officials was important in facilitating the study. The study objectives and relevance were explained to officials in relevant institutions, to aid ‘buy-in’ of the ideas, and permission to work was granted. Additionally, a clearer picture of the major actors in the dairy sector was achieved, as well as the challenges in gaining access to data for informed decision-making. 

Consent was obtained from the Ministry of Agriculture and Livestock Resources (MoALR), and the Ethiopian Meat and Dairy Industry Development Institute (EMDIDI) to identify and contact key informants within stakeholder clusters. These stakeholders were involved in the scoping and mapping process and in the stakeholders’ meeting and focus group discussions. Participants were informed of the project background, research purpose, and what would be involved, in the participant invite as well as verbally at the events. Written consent was not deemed necessary as the data collected was not relating to individuals or personally sensitive, but rather was regarding the needs of a much wider group (the dairy sector). Participant willingness and voluntary choice to attend was their consent to participate. 

### Stakeholder focus group discussions and interviews

Focus group discussions were conducted with the identified stakeholders in the three regions identified above, and in-depth interviews were held with key informants. 

In Addis Ababa a total of 17 focus group discussions and interviews were held, with one participant per interview and approximately three or four participants per focus group. A total of three discussions were held in Tigray and three were held in SNNPR. Focus groups and interviews were held at the workplaces of the relevant participants e.g. Ministry offices or at research institute premises. No participants declined to take part or dropped out. Focus groups and interviews lasted between one and two hours, with field notes taken to document the responses. Documentation and aggregation of common themes was conducted, with no further analysis at this stage. Focus group discussions and interviews were conducted in English and or Amharic. Interviews are included in Underlying data.

The purpose was to gain information on several issues:

•   major constraints and opportunities in the dairy sector

•   major stakeholders in the dairy sector, their current sources of data for important decision-making, and challenges they have in data availability and quality

•   strengths and weaknesses of existing data and data sources, and their implication for the development of the sector

•   mapping of the interaction between different stakeholders involved in collection, maintenance and dissemination of data in the sector

•   coordination of activities of the different actors involved in the collection and maintenance of data in the sector

•   central data storage and dissemination for dairy stakeholder issues, related data protection and property right in pooling data into a central hub and data sharing system

•   existing mechanisms of the dairy sector data sharing and dissemination, strengths and weaknesses and capacity building needs for better future services

•   key data needs for different dairy stakeholders

•   existing government policies on the collection, maintenance and dissemination of data for the dairy sector stakeholders, policy gaps (if any) and power vested on different institutions in this regard

•   existing capacity in data analysis and modelling to support informed interventions in the sector and who is doing what, and gaps in capacity

### Stakeholder workshop

A two-day workshop was held at the ILRI campus, Addis Ababa on 8 ^th^ and 9 ^th^ August 2018, involving 37 stakeholders representing the different actors in the Ethiopian dairy value chain, to discuss data needs. The workshop was structured in order to achieve a consensus of data needs while attempting to address any biases brought by the stakeholders; an itinerary was sent out to participants in advance, and discussions were based on open questions and gathering available data. A combination of talks given by participants and participant discussions were used in the workshop sessions. The workshop itinerary and a workshop report are presented in Extended data.

As well as the initial focus group discussions, additional group discussions were integrated in the workshop, designed to build on the stakeholder mapping and scoping processes carried out in the study. The key players and key issues regarding data needs that were identified in the mapping and scoping process contributed to the design and structure of the workshop.

The focus groups were made up of between four and seven participants in each of the five stakeholder groups (see Participant invite with workshop itinerary, Allan, 2022), and the discussions lasted 1–2.5 hours. Researchers present were Brian Perry, Yacob Aklilu, Getachew Legese, Solomon Hailemariam and Azage Tegegne. As before, field notes were taken to document the discussions, which were conducted in English and or Amharic. The workshop intended to identify and characterise current and future data needs by types, availability formats, level of detail, methods of dissemination, uptake and use, and institutional arrangements of handling, storing and accessing data, and the different roles of public and private sectors in decision-making processes. Stakeholders were classified into five groups, identifying their different data needs (the inputs and services stakeholders - feeds, genetics and health - were clustered for organisational purposes, bringing the eight clusters to five):

1. public sector policy and regulatory systems
2. inputs and service providers (public and private sector actors and NGOs)
3. private sector milk and dairy products producers
4. private sector milk and dairy product processors and marketers
5. dairy research and extension system actors

The groups were asked to identify the different data needs, consolidating them under the following headings:

•   What are the data needs?

•   Why do you need the data (purpose)?

•   In what format do you need the data (qualitative, quantitative or both)

•   In which categories is quality essential?

•   What level of resolution is required (geography, scale, region etc)

•   What are the major data gaps?

•   What is the time scale required for the data?

***Data needs.*** Information on data use and application was collected; how will you use the data; who else will use the data; will there be a cost to the data; how will it be stored; where will it be stored; how will the data be accessed?

***Data use and application***. Information was requested about data collection, quality control and management; how is/will the data be collected; how will the data be managed; how important is quality, and how will that be managed; how important is ease of use, and how is/will this be managed; how will data be disseminated and to whom; who will access the data; are there intellectual property issues?

***Institutional data use***. Institutional issues regarding data use, availability and access were also discussed; who will be responsible for responding to the data needs; is there adequate capacity to meet these needs, uses and access requirements; what should be the milestones for follow-up and actions following the workshop; what are the technology needs to ensure the data needs can be met; what are the communications needs required to ensure appropriate data access; what are the accountability needs?

### Synthesis of outcomes

Based on the outputs of the five stakeholder discussion groups, a synthesis of data needs by the different stakeholder clusters was conducted. All stakeholders were consulted on the findings and plans. Recommendations presented are those generated by the participatory processes i.e. are those of the participants.

## Results

### Literature review

The review of the literature highlighted the long-standing interest in the potential for greater productivity in the Ethiopian dairy sector, and identified many of the challenges to overcome these ( Guadu & Abebaw, 2016; Mihret *et al*., 2017; Ndambi *et al*., 2018; Yilma *et al.*, 2011). Challenges included the types of cattle, the limited production capacity of these cattle types, the vegan diet of 50% of the population, the poor coordination between producers, marketers and processors, and the inadequate feed resource for dairy animals. It also illustrated the lack of attention to the role of data in development and policy interventions by the government and development partners, as well as informing planning and decision-making for the different actors in the dairy sector. It also helped to understand the opportunities and constraints in the dairy sector, the major actors in the sector, their strengths and weaknesses, and interactions between different stakeholders.

### Stakeholder interactions

The process involved extensive dialogue and interaction, including travel by team participants. Stakeholder mapping and scoping identified the key players in different stakeholder categories, and the key issues regarding data needs; this helped to inform the ensuing structure and format of the workshop. Sector participants in Addis Ababa included two from government, five from research, one from feeds and two producers, amongst others. In Tigray there was one participant from the processing and two from the producer sectors, and in the SNNPR, there was one from each of the producers, feeds and processing sectors. A total of 37 participants attended the two-day stakeholder workshop. The participants included 32 males and five females.

***Data needs.*** Based on the outputs of the discussion groups, a synthesis of data needs by the different stakeholder clusters was conducted, and categorised under headings ( Table 1). A methodological approach was taken, which was refined during the workshop process.

**Table 1.** Synthesis of data needs by different stakeholder clusters and data categories, grouped under headings.

Category	Sub- category	Data needs
Dairy herd data		Livestock population numbers and category of dairy animals
		Dairy herd structure by species (including cattle, goats, camels), age group, blood type, location, livestock production system and agroecology
		Pedigree information
Production and reproduction		Major dairy farmers, location, herd size, daily milk production
		Milk production data at national, regional, zonal, woreda, household and individual animal level by breed of animals and production system
		Milk yield by breed and production system
		Milk composition by breed, stage of lactation and production system
		Lactation length
		Calving interval
		Service per conception
		Household milk utilization by location and production system: milk consumption at home by producers, milk processed at home, milk marketed – sold to processors and sold to raw milk distributors
Dairy input data needs	Feed	Major dairy feed producers by location and types of feed produced
		Type of feeds produced by different feed producers
		Volume of feed produced by the different actors (showing seasonality)
		Biomass yield of different forage species
		Quality – standard that different feed producers and distributors need to comply with
		Feed storage conditions
		Quality, availability and prices of different feed/forage types (including trends)
		Costs of transportation from points of feed production to the center/points of use
		Demand for feed by different dairy producers
		Major distribution channels for the different feed producers, volume of feed distributed through each channel by geographic location and season
		Availability and accessibility of the required quality and quantity of feed (by seasons and location)
		Economic minimum and optimum sizes of operation for production, and supply of different feed products
		Resources (finance, land, labour) needed for economic minimum and optimum size of different feeds production and their availability by location
		Access to resources such as land, finance and labour for forage production, feed grain production, feed processing and similar activities in the different regions
		Taxes levied on the different feed ingredients and mixed ration by region
		Incentives to promote feed production and processing
	Animal health services and veterinary drugs	History on prevalence of different diseases and parasites, by location, season and frequency of occurrence
		History on mortality/morbidity of animals, by location and cause
		Major disease diagnostic laboratories and service centers, by location
		Details of private and public veterinary service providers (by level of operation and location)
		Costs of the private and public health services
		Details of veterinary drugs, veterinary equipment (for clinics and laboratories) and manufacturing plants, importers, wholesalers, retailers (drug shops), by location and volume of operation
		Prices of veterinary services, drugs, equipment and consumables by supplier and location
		Incentives for veterinary service providers; vet drug, equipment and consumables manufacturers; importers; wholesalers and retailers
		Taxes on veterinary service providers and veterinary drug shops
	Genetic improvement	Details of public and private AI and bull service providers, by location (lists)
		Data on providers or AI services with sexed semen (their details including location, geographical coverage/ capacity)
		Quality of AI services including services per conception
		Data on the semen used for AI, including: pedigree information of the semen, blood level of semen, holders of semen with different specifications
		Sources of liquid nitrogen by location, production capacity, prices and their areas of operation
		Capacity of the available AI service and the cost of their services
		Details of suppliers of AI equipment and disposables (manufacturers, importers, wholesalers, retailers)
		Incentive packages to promote best AI services
		Taxes and other obligations
Dairy technology		Details on importers and distributors of dairy machinery (milking machines, milk processing machines, dairy laboratory machines etc), equipment, spare parts and consumable items by location, their volume of operation, product availability, prices etc
		Types of dairy machinery, equipment, spare parts and consumable items, their current prices, availability and sources
		Details of companies and individual experts providing technical supports including installation and maintenance services, training and technical advises on dairy machinery and equipment
Policy decisions and directives in dairy sector		Number and type of different actors, volume of operation and their geographic location
		Input need, availability and costs for different dairy actors by location
		Economic minimum and optimum levels of feed/forage production, processing and distribution; fabrication or import, wholesale and retail of veterinary drugs; provision of animal health and AI services; milk production, processing and distribution by geographic location
		Availability of finance, land, labour and technology for dairy input producers, processors and suppliers; milk producers, processors and distributors
		Land use plans of area identified for dairy development and comparative advantages of the different land use options
		Types and volume of imported dairy products, their sources, costs and wholesale and retail prices
		Major importers of dairy products
		Drivers of importing dairy products (quality issue, consumer preference, competitiveness)
		Quality standards and data on important parameters with respect to consumers safety (zoonotic diseases), hygienic and product handling requirements
		Taxes on the products for different actors in the dairy sector and gaps in these incentive packages for dairy investment
		Incentive packages for different actors in the dairy sector and gaps in these incentive packages for dairy investment
Dairy marketing		Major milk producers and suppliers to processors and consumers (raw milk), their supply volume by location, production system and season
		Major milk processing plants, their production capacity, product types and volume
		Prices for milk and milk products by location
		Quantity of raw milk produced and supplied to the different market channels by different actors (by geographic location) over years showing seasonal dimensions
		Consumer types and size for different milk and milk products over seasons and locations
		Demand volume for milk and different milk products by consumer type, season and location
		Consumer preference for different products, consumption size, and product quality considerations
		Imported milk and milk products, their source, quality, volume, source, consumer types, market share and prices
		Major marketing channels for different milk and milk products and product flow in each channel
		Product quality standards and consumer safety considerations

AI = artificial insemination.

***Defining data use and application***. All stakeholder groups indicated that the use of data was to make evidence-based decisions. More specifically, data were used for production planning, marketing and investment decisions, policy formulation, system harmonisation, allocation of resources to their best use, and improving the production and productivity in the dairy sector. They were also used for proper monitoring and evaluation by the regulatory system.

Forms of access to data were discussed and included the use of websites, mobile phone text messages, printed and electronic mass media, monthly bulletins and annual reports. In discussing the high costs associated with data collection, management and dissemination, subscriptions, memberships or direct payments were considered. 

***Exploring data collection, quality control and management***. It was agreed that data collection, quality control and management should follow standard procedures. The frequency of data collection depended on the types of data since some required regular collection and updating, depending on the required level of resolution (individual animal, district, zonal, regional and national levels). Some data required daily recording, for example, milk production by individual animals, whereas others only required recording on event occurrence, such as births and deaths. It was agreed that data collection required periodic updating, depending on its nature and purpose.

Different organisations were suggested as responsible for collection and management of dairy related data: Ministry of Agriculture and Livestock Resources Development; Central Statistical Agency; National Animal Genetic Resources Improvement Institute; National Dairy Board; universities; and an independent institution to be established for this purpose. Intellectual property rights would be assigned to the designated organisation and adherence to the data laws and general principles of seeking permission of owners/creators of data to acknowledge data sources in any form of use would apply.

***Exploring institutional arrangements***. It was reported that there was a lack of clarity in the institutional arrangements for generating, ownership and sustainable management of the dairy sector data, presenting a substantial challenge. A summary of the different institutions and stakeholders is shown in Table 2. 

**Table 2.** Summary of institutions and stakeholders in the dairy sector.

Stakeholder / role player in dairy sector	Directorate / Institute	Centre
Ministry of livestock/agriculture	Dairy development directorate	
	National Institute of Livestock Production (artificial insemination)	
	Directorate of veterinary services	National animal health diagnostic centre
	Ethiopian Institute of Agricultural Research	
	Regional Livestock Bureau	Regional veterinary laboratories
	Agricultural Transformation Agency (catalysing the GTP II and Livestock Master Plan transformative agenda	
Ministry of Health		
Ministry of Trade		
Ministry of Industry	Ethiopian meat and dairy industry development institute	
Ethiopian Investment Commission		
Ethiopian dairy producers association		
Ethiopian milk processors association		
Ethiopian animal feed industry association (EAFIA)		
Ethiopian veterinary association (EVA)		
Ethiopian Society of Animal Production (ESAP)		
World Bank		
Bill & Melinda Gates Foundation (BMGF)	Partnership for Artificial Insemination Delivery (PAID) project, via Land O’Lakes	
	African Dairy Genetic Gains (ADGG) project	
Smart Development Works (SNV) (International development agency engaged in Ethiopian dairy development)		
USAID (International development agency engaged in Ethiopian dairy development)		
Animal Health Research Institute (AHRI) (Ethiopian research institute)		
New Zealand Government Diary Development project (International agency engaged in dairy product development)		

There was no current viable institutional arrangement to address the data needs in the dairy sector, which was reported to be due, in part, to poor communication and linkage between institutions, as well as due to a lack of coherence and complementarity in the data categories generated by different organisations. 

Regarding the existing limitations in capacity – human, technological and institutional – participants suggested that an inventory of existing capacities be created, in order to understand and quantify the capacity requirements. A variety of initiatives had previously been tried to develop livestock information systems. Although these attempts had been unsuccessful, unused facilities existed within institutions, therefore it was suggested that a thorough inventory of the technological facilities be conducted, to help in proposing alternative capacity building interventions to address the existing gaps. It was indicated by some participants from the MoALR that there was the intention within the Ministry to establish their own livestock database, as well as a national dairy sector policy and the workshop was considered supportive and complementary to their work. The deficiencies of the Ministry, with respect to data, were acknowledged and praise was given to the pilot study and workshop for being timely and a legitimate response to the lack of appropriate and reliable data for planning, policy, investment and development activities in the dairy sector.

***Where a dairy sector database should be housed and managed***. A number of institutions were suggested to take the leading role in the hosting (development and management) of a dairy sector database, demonstrating a lack of unanimity. The National Animal Genetic Resources Improvement Institute (NAGII) was suggested, based on its experience and current responsibility in managing the database for livestock genetic resources. It was, however, suggested that the focus on livestock genetic improvement by the NAGII, may result in other issues such as input service, production, feeding, animal health, dairy production, processing and marketing, and consumption being overlooked. The MoALR was suggested, however there was concern that there may be a lack of capacity to develop and manage such datasets. Despite this concern, it was intended to establish a unit within the sector for livestock resources, to handle the dairy database.

It was suggested by the policy and regulatory stakeholders group that CSA and MoALR would be appropriate organisations for developing and handling a dairy sector database. The dairy inputs and services stakeholders group also suggested CSA, with strong technical support from MoALR. This would improve on the current sample survey data approach. The research and extension stakeholders group suggested MoALR should take sole responsibility with its regional bureaus. The dairy producers and processors suggested that a new independent organisation be established, such as a National Dairy Board, to work with government institutions to develop and handle a database. 

With regards to the establishment of a National Dairy Board, it is important to note that there have been several failed attempts over more than a decade. Such an initiative has also encountered hurdles in other countries of eastern Africa, even in countries with a much higher dairy production per cow ( Kurwijila & Bennett, 2011). Ironically, it is thought that inadequate data for policy makers to make evidence-based decisions resulted in failure to give permission for establishment of such an organisation. It has been observed that officials have questioned the purpose of establishing a dairy board in a country where there is no milk. As such, two important points were highlighted; i) there is a lack of reliable data on the performance of the dairy sector and the needs of the sector, in order to create the appropriate organisation, and ii) a variety of factors have held back the development of the sector, namely the lack of strong associations (producers cooperatives) that promote development, and the differing views on the role of government leadership.

## Discussion

There is scarce literature on the data needs of the dairy sector in Ethiopia, with most literature referring to the potential for dairy enterprise and those studies highlighting the challenges facing the sector ( Guadu & Abebaw, 2016; Mihret *et al*., 2017; Minten *et al*., 2020; Ndambi *et al*., 2018; Yilma *et al.*, 2011). A relatively recent study described the critical knowledge gap on these challenges, confirming the lack of research conducted on the difficulties and opportunities ( Didanna *et al.*, 2019). Similarly, a recent study at the International Livestock Research Institute (ILRI) highlighted a lack of market orientation, a lack of private sector investment, limited technical and financial capacity, weak market infrastructure and linkages across the value chain, and poor input and service delivery ( Gebreyohanes *et al.*, 2021). Minten *et al.* (2020) highlighted the requirement for ‘good data’ in order to understand patterns of transformation in the dairy sector. The recent development of Livestock Master Plans of different LMICs, in collaboration with ILRI, has emphasized the growing need for data, both to develop the plans, and to monitor interventions to meet targets set out ( https://www.ilri.org/livestock-master-plans).

The objective of this study was to establish the data needs of dairy sector stakeholders in Ethiopia, and to characterise them by data type, use and relevance to other stakeholders, as a fundamental priority for the sector as it seeks to respond to the rapidly growing demand for dairy products in Ethiopia. It is important to ensure efficient use of data for evidence-based decisions, and as such there is the necessity of the public good nature of the collected data.

An initial stakeholder scoping and mapping process was conducted in order to identify the dairy value chain actors. Focus group discussions and key informant interviews were held. A two-day workshop followed, with focus group discussions, building on the mapping process, to allow identification and characterisation of both current and future data needs by types, availability, formats, level of detail, methods of dissemination, uptake and use, and different roles of public and private sectors in decision-making processes. 

The methodology used in this study involved a process of deep engagement with the sector as a whole, and with representatives of different stakeholder clusters. This methodology could be adapted and utilised for similar undertakings in other countries, depending on existing realities on the ground. The methodology recognised the background of the team leader, as a facilitator with a long-standing understanding of Ethiopia’s agriculture and development, and the expertise of the Ethiopian scientists engaged in the study leadership team, and the focus on the needs of the Ethiopian dairy sector. It was important for local scientists to be involved, with robust understanding of the sector, the network of colleagues and institutions, and to give the study maximum ownership by Ethiopia; this was considered a key factor behind the success of the methodology used. All stakeholder groups indicated the requirement for both quantitative and qualitative data. Quantitative data were needed for planning and prioritisation, and qualitative data to provide indicators of trends. The groups indicated different methods of data collection to collect demand-driven and user-friendly data, available for use by different stakeholders in the dairy sector. Entrepreneurs (dairy input suppliers, service providers, producers, processors, distributors) required data for planning and timely decision making, in order to make their enterprises profitable. Researchers, investors, government and non-government development actors, academics, planners, policy-makers and the regulatory system required disaggregated data in the dairy sector, to accomplish their tasks. Overall, the needs across the different groups were found to be relatively homogenous, i.e. there was significant overlap across the groups *.* Some of the constraints and data needs ( Table 1) had been reported previously by Beyu (2016), including inadequate animal health services and high drug costs, inadequate quality feed supplies, poor productivity and genetics, and weak development of dairy co-operatives. Tschopp *et al*. (2021) also recently highlighted the lack of dairy productivity data in Ethiopia. It was generally accepted that quality data had monetary value, and access to it could incur a cost; however, many believed, at this stage of development, that as much data as possible should be made freely available. 

Huge data gaps were identified, in terms of sufficiency and consistency. There was a lack of disaggregation of breed information and a lack of clear productivity data. Animal health and disease data were considered to be poorly captured. Bottlenecks were discussed and included who should own and manage the data, linkages and communications between different data producers, coherence and complementarity amongst data producers, and accessibility of data. It was felt that the workshop provided a unique opportunity for stakeholder groups to discuss who could best serve as the designated body to address the data needs of the varying actors in the dairy sector.

It was thought that existing cooperatives are not effectively supporting the activity of their members. There is political motivation in cooperatives, with strong interference of government actors and challenges in the sector have not been addressed. Land access for the establishment of dairy farms was also discussed, with the acquisition of unoccupied land for dairying extremely challenging; government cannot provide enough land for dairy sector investment in agro-ecologically suitable, and economically feasible locations. As a result, shareholder agreements with smallholder farmers are often the only available option, converting land into dairy farming, or an out-grower scheme for forage production and related activities. Such challenges are constraining development of the sector and contributing to the bottlenecks in the realization of a National Dairy Board. Such a Board, however, could serve as a catalyst to explore some of the major challenges and develop viable solutions.

Sector-specific needs were identified and should be elevated in detail as they are currently somewhat generic, requiring further shaping within each stakeholder group. A proposal for the drafting of a national dairy sector policy was put forward by the dairy value chain office in the MoALR. It was recommended that this be followed up, ideally with funding to support the development, and to build on the products of this pilot study, in line with the Livestock Master Plan for Ethiopia.

Overall, based on the discussions, four alternative institutions were suggested to host (generate, manage, coordinate and communicate) the dairy database in Ethiopia, namely the National Animal Genetic Resources Improvement Institute (NAGII), the sector for livestock development under the MoALR, the Central Statistical Agency (CSA), and a proposed newly established National Dairy Board. Further study of these institutions is required, to understand in more detail their capacity and suitability to store, manage and dispense data to meet the different requirements in the country. Further data analyses and modelling could be of value in supporting evidence-based decision making for dairy sector development and will be explored by Livestock Data for Decisions (LD4D) data scientists, as one-off strategic technical support on design options.

It was recommended that every effort be put in to following up on the major outputs from the workshop, and that greater attention must be paid to building both horizontal and vertical links between stakeholder groups. There is, at present, a disconnect between the stakeholders, with no clear national leadership. It was proposed that the call for a National Dairy Board be reactivated and that such a body, independent of government but closely incorporating government, with the buy-in of private sector enterprises, would be likely to be seen as highly acceptable in the current political climate of Ethiopia. However, it would be useful to mitigate the risks of lack of political will and government capture, and overcome previous failures in the establishment of a National Dairy Board; this would likely entail some political economic analysis and recommended engagement with key actors based on the findings. The output from this study should form a sound base on which to develop a new submission to government for the creation of such a board. The dairy value chain office in the MoALR proposed the drafting of a national dairy sector policy and it is recommended that this suggestion be followed up, ideally with funding to support this development, and to build on the products of this study, in line with the Livestock Master Plan for Ethiopia.

It was recommended that all stakeholder groups should provide relevant and specific information to their constituents for informed decision making, to increase output, streamline operations, and minimise cost. Greater attention should be paid to the roles that different data play in informing and influencing proactive policies for the dairy sector. A national roll out of herd-specific data recording schemes for the emerging dairy sector was recommended, considering the example provided by the NAGII in the genetics component, and expanding it to encompass health, feed, and other input services to provide effective evidence-based extension services to dairy farmers.

## Limitations

The under-representation of women in the workshop resulted in the dominance of male participants in the discussions, which does not help to redress the balance in establishing the priorities of both male and female dairy farmers. There was also the potential for the most well-known/articulated stakeholders to dominate discussions. Attempts to mitigate this potential bias were made by recruiting as wide a range of participants and stakeholders as possible.

## Conclusion

The study provided a timely discussion with and between diverse stakeholders involved in the dairy industry in Ethiopia, including policy makers, producers, processors, service providers, and researchers. The products on data needs represented a broad set of groups within each of these categories, including regional perspectives in Tigray and SNNPR.

From the study, several potential organisations were suggested to host and manage a national dairy database. Importantly, the establishment of a National Dairy Board was strongly endorsed. The long-term sustainability of any new institutional arrangements need to be considered in addition to the functionality and business approach advocated by many of the stakeholders. It would be worth conducting a SWOT analysis on the establishment of a Dairy Board, to clearly identify the strengths, weaknesses, opportunities and threats, and engaging multiple stakeholders in this process. It was recommended that stakeholder links be established, sector-specific data needs be elevated to higher detail, and a national roll out of herd-specific data recording schemes was called for, to allow for effective evidence-based policies and decision making.

## Data availability

### Underlying data

Harvard Dataverse: Replication Data for: A pilot study of the data demands of different stakeholders for the future Ethiopian dairy sector https://doi.org/10.7910/DVN/PMKXOQ" https://doi.org/10.7910/DVN/PMKXOQ ( Allan, 2022)

This project contains the following underlying data:

- FINAL_Interviews with Stakeholders.pdf

Due to the full data containing identifiable information, it has not been made publicly available. Researchers in a similar field can request further details of the interviews and discussions from the corresponding author, prof.brianperry@gmail.com.

### Extended data

Harvard Dataverse: Replication Data for: A pilot study of the data demands of different stakeholders for the future Ethiopian dairy sector https://doi.org/10.7910/DVN/PMKXOQ" https://doi.org/10.7910/DVN/PMKXOQ ( Allan, 2022)

This project contains the following extended data:

- Participant invite with workshop itinerary.pdf
- Checklist for Ethiopian Dairy Sector Needs Workshop.pptx
- Anonymous_Report_Dairy data needs workshop.pdf

Data are available under the terms of the Creative Commons Zero "No rights reserved" data waiver (CC0 1.0 Public domain dedication).
