# Supplementary material for: A multi-stakeholder participatory pilot study of the data demands of the future Ethiopian dairy sector
Source: Gates Open Res. 2022 Aug 2;6:51. Originally published 2022 Apr 22. [Version 3] doi: 10.12688/gatesopenres.13594.3 (PMC9296832; doi:10.12688/gatesopenres.13594.3)
Supplement: Peer review history

# Peer review: Reviewer response for version 3

**DOI:** 10.21956/gatesopenres.15035.r32351

**Published:** 2022-08-04

**Authors:** Ulf Magnusson

**Affiliations:** 1 Department of Clinical Science, Swedish University of Agricultural Science, Uppsala, Sweden

I appreciate the responses from the authors.

Is the work clearly and accurately presented and does it cite the current literature?

Yes

If applicable, is the statistical analysis and its interpretation appropriate?

Not applicable

Are all the source data underlying the results available to ensure full reproducibility?

Yes

Is the study design appropriate and is the work technically sound?

Yes

Are the conclusions drawn adequately supported by the results?

Yes

Are sufficient details of methods and analysis provided to allow replication by others?

Partly

Reviewer Expertise:

Veterinary medicine - infectious diseases and reproduction, sustainable livestock production in LMICs

I confirm that I have read this submission and believe that I have an appropriate level of expertise to confirm that it is of an acceptable scientific standard.

# Peer review: Reviewer response for version 2

**DOI:** 10.21956/gatesopenres.14943.r32208

**Published:** 2022-07-19

**Authors:** Gizat Almaw

**Affiliations:** 1 Animal Health Institute, Sebeta, Ethiopia

Overall the study is sound and relevant in that it has identified an important gap/s in the dairy sector of Ethiopia. However, suggestions are forwarded to improve the paper.

 Introduction

 The argument/hypothesis for conducting this study needs to be stronger. The authors have provided adequate background on the challenges Ethiopia’s dairy sector is facing. However, how the data need and application fit into these challenges is not detailed/addressed. Also good the authors provide background on the experiences of data use and application elsewhere with due emphasis on LMICs and present this in perspective with the Ethiopia dairy sector. The CSA of Ethiopia every year conducts an agricultural sample survey including livestock and the data is freely available. The limitation/s of this database with regard to the dairy sector needs to be also highlighted. The scope of the study needs to be clear. Does it apply to all dairy farming systems including the pastoral production system? From the identified clusters it looks pastoral system was not part.

 Study design

 The approach for stakeholder mapping and cluster identification needs additional clarification. Was it purposive or convenient? Was the geographic spread following the milk sheds or other criteria? Only two regional states were represented. What was the reason for this? Good authors provide additional information for the readers about how stakeholders/areas were selected.

Is the work clearly and accurately presented and does it cite the current literature?

Yes

If applicable, is the statistical analysis and its interpretation appropriate?

Not applicable

Are all the source data underlying the results available to ensure full reproducibility?

Yes

Is the study design appropriate and is the work technically sound?

Yes

Are the conclusions drawn adequately supported by the results?

Yes

Are sufficient details of methods and analysis provided to allow replication by others?

Yes

Reviewer Expertise:

Bovine Tuberculosis

I confirm that I have read this submission and believe that I have an appropriate level of expertise to confirm that it is of an acceptable scientific standard.

# Response

**Published:** 2022-07-22

**Authors:** Fiona Allan

**Affiliations:** Royal (Dick) School of Veterinary Studies, UK

Dear Reviewer,

 We sincerely thank you for your time and effort in providing expert assessment of our manuscript.  There are constructive points raised which we have considered and hope that we have addressed suitably.

 Responses to comments are listed in italic font below, with additions and amendments highlighted in bold.

1. Introduction:  The argument/hypothesis for conducting this study needs to be stronger. The authors have provided adequate background on the challenges Ethiopia’s dairy sector is facing. However, how the data need and application fit into these challenges is not detailed/addressed.
*The data needs role is stated with regard to the multiple targets set by the Livestock Development plan (last paragraph of Introduction).*
2. Also, could the authors provide background on the experiences of data use and application elsewhere with due emphasis on LMICs and present this in perspective with the Ethiopia dairy sector.
*We will add to the manuscript, at the end of the first paragraph of the Discussion: **The recent development of Livestock Master Plans of different LMICs, in collaboration with ILRI, has emphasized the growing need for data, both to develop the plans, and to monitor interventions to meet targets set out*** ( https://www.ilri.org/livestock-master-plans).
3. The CSA of Ethiopia every year conducts an agricultural sample survey including livestock and the data is freely available.  The limitation/s of this database with regard to the dairy sector needs to be also highlighted.
*We will add to the manuscript, in paragraph 2 of ‘Where a dairy sector database should be housed and managed’, after ‘…also suggested CSA’: **This would improve on the current sample survey data approach.***
4. The scope of the study needs to be clear. Does it apply to all dairy farming systems including the pastoral production system? From the identified clusters it looks pastoral system was not part.
*While the study reviews the national needs, the available time and funds limited the geographical scope of the in-depth analysis. Pastoral systems were not visited specifically. This was potentially compensated by the wide range of stakeholder engagement in the discussions and workshop. One of the authors (Yacob) has extensive experience in pastoral systems, and brought that knowledge (and institutional connections) to the table.*
5. Study design: The approach for stakeholder mapping and cluster identification needs additional clarification. Was it purposive or convenient?  Was the geographic spread following the milk sheds or other criteria?  Only two regional states were represented. What was the reason for this? Good authors provide additional information for the readers about how stakeholders/areas were selected.
*The study was purposive, as set out in the methodology. The geographic spread covered a) the major consumption centre, the Addis Ababa region), b) one of the recognised production areas (SNNPR), and c) Tigray, an example of regional differences in demand and production*
*We will add to the manuscript, in Study Design: “The **purposive **study….”*

# Peer review: Reviewer response for version 2

**DOI:** 10.21956/gatesopenres.14943.r32126

**Published:** 2022-07-19

**Authors:** Ulf Magnusson

**Affiliations:** 1 Department of Clinical Science, Swedish University of Agricultural Science, Uppsala, Sweden

Studies like the current one – a multi-stakeholder approach to identify data demands for development of the livestock sector in low-income countries – are dearly needed and applaudable. Given the inherited qualitative aspect of such studies, they are however at risk for biases, particularly “the usual-suspect-syndrome” in which the most well-known/articulated stakeholders dominate. In the current study have the authors, however, made reasonable efforts to mitigate this bias. Still, it would be appropriate to mention this risk under the “Limitations”-heading together with the gender bias.

 In the following there are some minor suggestions for the authors’ consideration that may improve the manuscript:

- It might not be understood for all readers of the introduction that cross-bred dairy cows are more productive (if appropriately fed and kept healthy) than the indigenous breeds.
- In the last sentence of the first paragraph, consider replacing “reproductive challenges” with “reproductive disorders” to avoid repeating “challenges”.
- Under “Study design”, is it relevant to tell about the first author’s 50 years of experience in Ethiopia as the team is a mix of Ethiopian and UK nationals?
- Under “Literature review”: the documents made available in Ethiopia; what kind?  Governmental reports, other grey literature – is it possible to specify?
- “Stakeholder scoping and mapping”: The list of stakeholder cluster seems to be a mix of kinds of stakeholders (e.g. Gov) and topics (e.g. feed) – is it possible to make this more systematic/consistent?
- Likely it would be helpful for the reader to have a diagram that provides an overview of the flow of the information and discussions feeding into the conclusions of the study. That is; literature review, focus group discussions, interviews, and the stakeholder workshop including the number and categories of persons approached/involved.

Results:

- Literature review, aren’t the challenges already presented in the very first paragraph of the MS (i.e. the references Yilma and Gaudu & Abebaw)? It becomes a kind of circular writing.
- Stakeholder interaction: is the travel by the team relevant to state – isn’t it the face-to face dialogues that are important?
- Data needs: What does the “methodological approach” mean?
- Table 1. “stakeholder clusters”, again this seems to be a mix of topics/issues and other aspects in the columns “Category” and “Subcategory”, not really stakeholders like Gov, NGOs, farmers organisations, commercial suppliers… Moreover, it is doubtful that the shading of every second row adds clarity to the table.
- Table 2. Several of the stakeholders’ names are very descriptive regarding their role, however, others – the foreign – are not. It would be helpful if the latter could be given some explanation of their role (like for BMGF).

Discussion

- 3 ^rd^ paragraph, isn’t this very much a repetition of the result and material and methods section?
- Rightly, data management and availability are elaborated on by the authors in several parts of the discussion. Based on the experience from other countries this might be a bottleneck for efficient use of data for evidence-based decisions, so it is recommended that the authors stress the necessity of the public goods nature of the collected data.

Is the work clearly and accurately presented and does it cite the current literature?

Yes

If applicable, is the statistical analysis and its interpretation appropriate?

Not applicable

Are all the source data underlying the results available to ensure full reproducibility?

Yes

Is the study design appropriate and is the work technically sound?

Yes

Are the conclusions drawn adequately supported by the results?

Yes

Are sufficient details of methods and analysis provided to allow replication by others?

Partly

Reviewer Expertise:

Veterinary medicine - infectious diseases and reproduction, sustainable livestock production in LMICs

I confirm that I have read this submission and believe that I have an appropriate level of expertise to confirm that it is of an acceptable scientific standard.

# Response

**Published:** 2022-07-22

**Authors:** Fiona Allan

**Affiliations:** Royal (Dick) School of Veterinary Studies, UK

Dear Reviewer,

 We sincerely thank you for your time and effort in providing expert assessment of our manuscript.  There are constructive points raised which we have considered and hope that we have addressed suitably.

 Responses to comments are listed in italic font below, with additions and amendments highlighted in bold.

1. Studies like the current one – a multi-stakeholder approach to identify data demands for development of the livestock sector in low-income countries – are dearly needed and applaudable. Given the inherited qualitative aspect of such studies, they are however at risk for biases, particularly “the usual-suspect-syndrome” in which the most well-known/articulated stakeholders dominate. In the current study have the authors, however, made reasonable efforts to mitigate this bias. Still, it would be appropriate to mention this risk under the “Limitations”-heading together with the gender bias.
*This comment is understood, and yes, the authors made considerable efforts to mitigate this potential bias.  We will add to the manuscript, in the Limitations paragraph: *
***There was also the potential for the most well-known/articulated stakeholders to dominate discussions.  Attempts to mitigate this potential bias were made by recruiting as wide a range of participants and stakeholders as possible.***
2. It might not be understood for all readers of the introduction that cross-bred dairy cows are more productive (if appropriately fed and kept healthy) than the indigenous breeds.
*We will add to the manuscript, in the Introduction paragraph 3, after ‘These cattle also have a short lactation length of about 150 days (Tegenge, 2018). *
***Cross bred cattle, in comparison, produce 10-15 litres per day and have a lactation period of around 300 days (Zilstra et al., 2015).***
3. In the last sentence of the first paragraph, consider replacing “reproductive challenges” with “reproductive disorders” to avoid repeating “challenges”.
*We will amend this in the manuscript*
4. Under “Study design”, is it relevant to tell about the first author’s 50 years of experience in Ethiopia as the team is a mix of Ethiopian and UK nationals
*Interesting question! The other UK nationals are from SEBI, specialising in data needs, the first author was responsible for study design and interaction with different institutions, for which a long-standing knowledge of the country was seen to be an advantage, and endorse credibility. *
*We will **replace 50 years with long-standing experience.***
5. Under “Literature review”: the documents made available in Ethiopia; what kind?  Governmental reports, other grey literature – is it possible to specify?
*We will add to the manuscript ‘documents were made available to the team in Ethiopia, **including non-published governmental and grey literature reports***
6. “Stakeholder scoping and mapping”: The list of stakeholder cluster seems to be a mix of kinds of stakeholders (e.g. Gov) and topics (e.g. feed) – is it possible to make this more systematic/consistent?
*We believe the manuscript does list in a clear systematic manner; Producers, processers, services (health, feed, breeding), government agencies, etc.*
7. Likely it would be helpful for the reader to have a diagram that provides an overview of the flow of the information and discussions feeding into the conclusions of the study. That is; literature review, focus group discussions, interviews, and the stakeholder workshop including the number and categories of persons approached/involved.
*As above, we feel this information is already presented in a systematic manner*
8. Results: Literature review, aren’t the challenges already presented in the very first paragraph of the MS (i.e. the references Yilma and Gaudu & Abebaw)? It becomes a kind of circular writing.
*We were advised by an editor to cite some of the literature as findings/results of the literature review.*
9. Stakeholder interaction: is the travel by the team relevant to state – isn’t it the face-to face dialogues that are important?
*Travel is relevant in that it describes regional differences in stakeholder categories, and in regional differences in importance of the dairy sector.  Travel was required in order to establish the face-to-face dialogues.*
10. Data needs: What does the “methodological approach” mean?
*This was in response to Reviewer #1 who asked for clarification on whether the synthesis was part of methods or impromptu i.e. the approach was methodological, with refinement.*
11. Table 1. “stakeholder clusters”, again this seems to be a mix of topics/issues and other aspects in the columns “Category” and “Subcategory”, not really stakeholders like Gov, NGOs, farmers organisations, commercial suppliers… Moreover, it is doubtful that the shading of every second row adds clarity to the table.
*We will add to table caption, “…different stakeholder clusters **and data categories..”**.  We can request the shading be removed (this was added by the journal).*
12. Table 2. Several of the stakeholders’ names are very descriptive regarding their role, however, others – the foreign – are not. It would be helpful if the latter could be given some explanation of their role (like for BMGF).
*We will add a basic description to the stakeholders:*
*SNV **(International development agency engaged in Ethiopian dairy development)***
*USAID **(International development agency engaged in Ethiopian dairy development)***
*AHRI **(Ethiopian research institute)***
*New Zealand Government Dairy Development project **(International agency engaged in dairy product development)***
13. Discussion:3 ^rd^ paragraph, isn’t this very much a repetition of the result and material and methods section?
*This was intended as a brief reminder/summary of the study.*
14.  Rightly, data management and availability are elaborated on by the authors in several parts of the discussion. Based on the experience from other countries this might be a bottleneck for efficient use of data for evidence-based decisions, so it is recommended that the authors stress the necessity of the public goods nature of the collected data.
*This can be stressed.  We will add to the end of the second paragraph of the discussion: **It is** **important to ensure efficient use of data for evidence-based decisions, and as such, there is a necessity of the public good nature of the collected data.***

# Peer review: Reviewer response for version 2

**DOI:** 10.21956/gatesopenres.14943.r32125

**Published:** 2022-06-08

**Authors:** Thomas Randolph, Amos O. Omore

**Affiliations:** 1 International Livestock Research Institute, Nairobi, Kenya

We appreciate the good effort by the authors to address a number of our concerns and suggestions. The main outstanding suggestion is to provide an evaluation or lessons learned regarding the participatory process. In their response, the authors note additional limitations related to political sensitivities to explain why some of the originally targeted information to permit more detailed analysis of data needs and the roles of different actors could not be captured during the process: this is a critical lesson to temper the expectations of anyone planning to replicate such a process and so merits comment.

Is the work clearly and accurately presented and does it cite the current literature?

Partly

If applicable, is the statistical analysis and its interpretation appropriate?

Not applicable

Are all the source data underlying the results available to ensure full reproducibility?

Yes

Is the study design appropriate and is the work technically sound?

Yes

Are the conclusions drawn adequately supported by the results?

Partly

Are sufficient details of methods and analysis provided to allow replication by others?

Yes

Reviewer Expertise:

Randolph: Animal health and policy economics; Omore: Veterinary epidemiology and economics

We confirm that we have read this submission and believe that we have an appropriate level of expertise to confirm that it is of an acceptable scientific standard.

# Peer review: Reviewer response for version 1

**DOI:** 10.21956/gatesopenres.14865.r31959

**Published:** 2022-05-09

**Authors:** Thomas Randolph, Amos O. Omore

**Affiliations:** 1 International Livestock Research Institute, Nairobi, Kenya

**Review of A pilot study of the data demands of different stakeholders for the future Ethiopian dairy sector. Perry et al.**

**Approved with Suggestions:** The reviewers believe the paper has academic merit but have asked for a number of small changes to the article, or specific, sometimes more significant revisions.

 Questions specific to a research article:

- Is the work clearly and accurately presented and does it cite the current literature?

Partly. The article includes a review of the literature that cites what is currently available regarding the sector, including unpublished reports that are relevant. However, it would benefit by acknowledging literature regarding the methodology in the context of information sciences. An additional reference to cite is the 2011 FAO report on ‘Dairy Development Institutions in East Africa - Lessons learnt and options’ with experiences on roles of dairy boards in the region that may be relevant as benchmarks in facilitating dairy knowledge and information systems. ^1^ The authors could perhaps go further to look at performance of dairy boards in other LMICs in this respect (e.g., India?). The experiences might help the authors to re-evaluate the risk associated with the key recommendation of (re-) establishing a dairy board in Ethiopia to play similar roles. I guess the report is available from FAO’s repository.

- Is the study design appropriate and does the work have academic merit?

Yes, on both counts. The article describes a participatory process for assessing data needs that is appropriate for the objective of strengthening sectoral capacity within a country. It has academic merit in terms of describing a process relevant elsewhere, providing a novel focus on an information sciences issue relevant to development. It could be strengthened from an academic perspective in terms of (1) establishing its fit in information sciences; (2) evaluation of strengths and weaknesses – as noted in the methods section, but not followed up in the results or discussion sections – or lessons learned during the study, beyond the results achieved specific to the Ethiopian context; and (3) displaying more fully the results consistent with the study objective, especially with respect to relevance of different types of data to the various stakeholders.

- Are sufficient details of methods and analysis provided to allow replication by others?

Yes, the method in terms of process is well described, including the development of the data collection instrument in the form of an interview check-list. The study basically stops at the collection of data and the participatory review and could offer more in terms of how the data can be effectively analysed to inform next steps and what principles to apply to such an analysis.

- If applicable, is the statistical analysis and its interpretation appropriate?

Not applicable.

- Are all the source data underlying the results available to ensure full reproducibility?

Yes, the interview check-list and stakeholder workshop invitation are provided. Could be strengthened by: (1) a more detailed description of how the individual workshop sessions were conducted; and (2) referencing a workshop report that would serve as confirmation and a record of the recommendations agreed by the participants.

- Are the conclusions drawn adequately supported by the results?

Partly. The conclusions report the general recommendations emerging from the participatory process and are consistent with the account provided by the study authors. It is not clear, though, whether the recommendations represent the explicit recommendations representing the consensus of the workshop participants – which would benefit from reference to a workshop report – or also the recommendations of the study authors: it would be useful to clarify this point. Also, importantly, the discussion and conclusions do not address risks associated with the recommendations especially in terms of repeating previous failures, including ‘who pays, who benefits?’ and potential for un-intended consequences (as have occurred in neighbouring Kenya). In particular, the risk of lack of political will – often a threat – merits highlighting going by the experiences from Tanzania and Kenya. The stakeholder ambition to have a strong autonomous dairy board in Tanzania that could play this role was thwarted at the last minute without further reference to them. Clauses in the dairy bill that envisaged autonomy and a stakeholder driven dairy board were quashed by the government before its enactment, resulting in a body that is essentially a government agency. This is the reason stakeholders started to pursue a different private sector driven approach – the Dairy Development Forum - to fulfil their initial ambitions. More attention should be given to why the proposed ambitious data collection systems would be expected to achieve wider uptake and be sustained.

**Review**

 This study addresses the critical need for data to inform dairy development in Ethiopia by describing a process for assessing those needs and related capacities across the range of relevant actors in the dairy sector. In addition to confirming stakeholder recognition of the need for such data, the process facilitated stakeholder identification of a strategy to begin addressing the needs by establishing a national dairy board that could offer a role in taking responsibility for coordinating the various interests and capacities across the sector, recognizing the risk that it could be captured by government interests. The contribution of the study is therefore to provide a methodology and an example of its application as a participatory process to strengthen sectoral data generation and management that can be replicated for other contexts, whether geographically or for other sectors.

 The study would be further strengthened from addressing four key areas:

- The study approaches the data issue as a development problem, using a review of literature to justify the data issue as a major constraint to dairy development in Ethiopia. It should also recognize that the data issue falls into the domain of information sciences – not necessarily to do an extensive literature review, but to at least acknowledge there is a relevant literature there, too.
- The objective of the study is “to establish the data needs of dairy sector stakeholders in Ethiopia, and to characterize them by data type, use and relevance to other stakeholders, as a fundamental priority for the sector as it seeks to respond to the rapidly growing demand for dairy products in Ethiopia.” Data presented in the article is limited to a list of data needs and a list of dairy sector stakeholders. These are very useful as baseline information but do not cover the more specific data that appears to have been collected regarding data type, use and relevance to the various stakeholders. A summary of these findings would be expected to be presented as part of the article to serve as a critical starting point for the follow-up analysis and actions recommended by the study. If this information was indeed collected, either Table 1 or Table 2 could be expanded to provide more detail about the type of data (scope or resolution, frequency) and relevance to each stakeholder group.  Also consider elaborating the purpose bullets in the methods section on a) ‘strengths and weaknesses of existing data and data sources’, and b) ‘capacity building needs for better future services’ where it appears the role of the dairy board is envisaged.
- The Results section of the article mentions that “there have been several failed attempts” presumably referring to establishing a national dairy board. One of the reviewers is familiar with such a failed attempt a decade earlier as part of the USAID funded ILRI/ASARECA work to harmonize dairy policies in eastern Africa. Ethiopia was dropped half-way through the process because stakeholders could not hold a national meeting to consider proposals if such a consultation was not driven by government. This suggests that there is a significant risk of history repeating itself if the recommendation to reactivate a national dairy board is followed blindly. It would therefore be important to characterize this risk more fully and consider the factors that could undermine or strengthen the sustainability of both the dairy board and the various data collection efforts proposed. There would be value to some additional discussion of the costs and benefits of the dairy board and the data collection efforts from the perspectives of the different stakeholder groups to qualify what otherwise might be viewed as a high-risk recommendation. An additional reference to cite in this regard is the 2011 FAO report on ‘Dairy Development Institutions in East Africa - Lessons learnt and options’ with experiences on roles of dairy boards in the region that may be relevant as benchmarks in facilitating dairy knowledge and information systems.  The authors could perhaps go further to look at performance of dairy boards in other LMICs in this respect (e.g., India?). The experiences might help the authors to re-evaluate the risk associated with the key recommendation of (re-) establishing a dairy board in Ethiopia to play similar roles.
- It would be useful if to clarify which analyses and recommendations were generated by the participatory process during the workshop and which were provided by the authors themselves.

More specific suggestions:

**Title**

 The authors may want to consider having the participatory process dimension of the study captured more explicitly in the title and objectives.

**Introduction**

 It would be helpful to explain apparent inconsistencies in bullets in the Introduction and later text between:

- “Milk production is predicted to increase from 167 million to 1,490 million litres” and “Combined interventions are predicted to result in a 93% increase in milk production, from 4,132 to 7,967 million litres”

- “GDP contribution from dairy will increase from Ethiopian Birr (ETB) 1.1 billion to 10.0 billion” and “Increased contribution of cow milk to GDP from ETB 28 billion to 52.9 billion”

Would help to clarify what is meant by “Combined interventions” in the sentence quoted in the preceding bullet.

 The initial Purpose statement “By establishing the data needs of stakeholders, priorities and recommendations could be made for how best to develop the dairy sector of Ethiopia” looks to be more about priorities and recommendations for how data can be generated and managed that would best support dairy sector development rather than directly deciding the development strategy.

 Constraints from Yilma *et al *2011 are cited in one sentence, then the following sentence cites the FAO (Yilma *et al* 2011) study as if it is a new reference being introduced. It would make sense to consolidate the information from the FAO study in one sentence.

**Methods**

 For “Clusters were identified by defining the dairy value chains in Ethiopia”, suggest something like “stakeholder clusters were identified according to key roles undertaken by the various actors with dairy value chains in Ethiopia.”

 “Contact details were provided by the stakeholders in each of the clusters” could be interpreted as raising an ethics concern, especially given the subsequent GPDR requirements. Could perhaps add that as an issue to help justify the appropriateness of the ethics approval.

 “Local authors provided lists of suitable participants, based on their local knowledge.” Consistent with other statements about potential bias, it would be useful to explain whether there was potential for bias to be introduced here.

 Eight clusters listed initially, but workshop refers to five stakeholder groups: would be helpful to note and explain the change.

 The structure is a bit confusing after “The groups were asked to identify the different data needs, consolidating them under the following headings”: some of the bullets are repeated in the following paragraphs, some are not. Would be useful to clarify the distinction between the bullets and three subsequent paragraphs.

 “Synthesis of outcomes: …. Relevant policy makers were consulted on the findings and plans.” Merits brief explanation why policy makers were targeted rather than all stakeholders.

**Results**

 Stakeholder interactions: starts by repeating information relevant to the methods section, which could be reduced. Would benefit from more evaluation of how it was implemented compared to the design/plan, e.g., adaptations, challenges.

 Composition data are presented for the pre-workshop interviews; it would be useful to have workshop participant numbers also presented by cluster/stakeholder group.

 Data needs: not clear if synthesis was part of methods or impromptu; it would be useful to have more clarity of any changes made in the method and what the results revealed. Alignment of this and subsequent paragraphs with the headings and the bullets in the methods section may also improve clarity

 Table 1: It is stated in the text that “The objective of this study was to establish the data needs of dairy sector stakeholders in Ethiopia, and to characterize them by data type, use and relevance to other stakeholders, as a fundamental priority for the sector as it seeks to respond to the rapidly growing demand for dairy products in Ethiopia.” Results are not presented, though, regarding the “relevance to other stakeholders”. Table 1 could be expanded to include this information with extra columns noting which stakeholder group may have expressed the need and which other stakeholder groups find it relevant – as critical information when designing data collection/coordination mechanisms. Or in Table 2, indicated which data categories were considered a priority for each stakeholder.

 If any information was collected on how to overcome weaknesses in the institutions in playing their roles, this should also be reported.

 Would be useful to cite some examples of “evidence-based decisions” and by which stakeholder.

 Data use: “They were also used for proper monitoring and evaluation by the regulatory system” Would be useful to cite a couple of examples of what this might refer to.

 Data collection: “… and general principles of seeking permission of owners/creators of data to acknowledge data sources in any form of use.” Incomplete, missing something like ‘would apply’ or ‘would need to be respected’.

 Institutional arrangements Table 2: While the actors are listed, it would be useful to have a sense of the role they seem themselves or others see them playing within the sector (and were these the same?), and possibly clustering them in the table accordingly. Presumably, this type of information would have been collected and validated during the workshop. It would also be useful to report in the table any relevant strengths and weaknesses for the individual institutions identified by the stakeholders.

 There appears to be repetition between the paragraph entitled ***Where a dairy sector database should be housed and managed*** and the second paragraph preceding it: both discuss candidate institutions for housing the database and so should be merged.

 Housing a database: “…it was intended to establish a unit within the sector for livestock resources, to handle the dairy database.” Would be good to clarify who has the intention and if the MoALR, would they establish it within the ministry or just somewhere ‘within the sector’?

 “… there have been several failed attempts” Presumably refers to establishing some type of a national dairy board, but would be good to specify for sake of clarity. Also would be useful to clarify who made the attempts.

 “… a variety of factors have held back the development of the sector, namely strong associations (producers cooperatives) that support development, and challenges in access to land.” Suggests strong associations have held back development; or was it meant to be ‘lack of strong associations'?

**Discussion**

 “Sector-specific needs were identified and should be elevated in detail as they are currently somewhat generic, requiring further shaping within each stakeholder group” Not sure what ‘elevated in detail’ means.

 “… the sector for livestock development under the MoALR” What is a sector under a ministry? Sounds like a unit or equivalent to the other agencies.

 “… a newly established National Dairy Board” implies it already exists. Should it be: ‘proposed’?

 “Further data analyses and modelling could be of value in supporting evidence-based decision making for dairy sector development and will be explored by Livestock Data for Decisions (LD4D) data scientists.” Would be useful to clarify how this fits with the focus on creating sustainable domestic institutional capacity – should it be more explicitly framed as a one-off strategic technical support or input for designing ?

 Some of the workshop recommendations might be interpreted as ‘wishlist’ expressions of ‘want’ as sometimes come out of such workshops. The Discussion is meant to be more analytical, so is it possible to help the reader distinguish?

 Focus in the Discussion is on establishing a new entity, but with little attention or analysis of its potential to be sustained. It would benefit from more discussion around the information collected/results and action needed that would support addressing sustainability issues during the design, especially given the comment made about previous failed attempts.

**Conclusion**

 “… reactivation of a national dairy board was strongly endorsed.” This implies one had existed; it would be useful to clarify whether one previously established that was being reactivated, or whether it was the proposal for one that was being reactivated.

 While the recommendation to re- establish a national dairy board is ‘likely to be seen as acceptable in the current political climate’ as noted by the authors, it would be useful to consider a recommendation that would mitigate the risks of lack of political will and government capture and overcome previous failures to establish such a body. This might entail some political economy analysis and recommended engagement with key actors based on the findings.

 Again, the Conclusion seems to assume ‘build it and they will come’, i.e. assuming roll-out will automatically ensure uptake and sustainability. It would be useful to flag any risks and highlight what is needed to avoid repeating earlier failed attempts.

Is the work clearly and accurately presented and does it cite the current literature?

Partly

If applicable, is the statistical analysis and its interpretation appropriate?

Not applicable

Are all the source data underlying the results available to ensure full reproducibility?

Yes

Is the study design appropriate and is the work technically sound?

Yes

Are the conclusions drawn adequately supported by the results?

Partly

Are sufficient details of methods and analysis provided to allow replication by others?

Yes

Reviewer Expertise:

Randolph: Animal health and policy economics; Omore: Veterinary epidemiology and economics

We confirm that we have read this submission and believe that we have an appropriate level of expertise to confirm that it is of an acceptable scientific standard, however we have significant reservations, as outlined above.

## References

[1] : Dairy development institutions in East Africa - Lessons learned and options. *Food and Agriculture Organization of the United Nations* .2011;

# Response

**Published:** 2022-05-20

**Authors:** Fiona Allan

**Affiliations:** Royal (Dick) School of Veterinary Studies, UK

                                                                                                                20 ^th^ May 2022

 Dear Reviewers,

 We sincerely thank you for your time and effort in providing an expert assessment of our manuscript.  There are many constructive points raised that we have considered and hope that we have addressed suitably.

 Response to comments are listed in italic font below, with additions and amendments highlighted in bold.

**Review of A pilot study of the data demands of different stakeholders for the future Ethiopian dairy sector. Perry *et al.***

**Approved with Suggestions:** The reviewers believe the paper has academic merit but have asked for a number of small changes to the article, or specific, sometimes more significant revisions.

 Questions specific to a research article:

- Is the work clearly and accurately presented and does it cite the current literature?

Partly. The article includes a review of the literature that cites what is currently available regarding the sector, including unpublished reports that are relevant. However, it would benefit by acknowledging literature regarding the methodology in the context of information sciences. An additional reference to cite is the 2011 FAO report on ‘Dairy Development Institutions in East Africa - Lessons learnt and options’ with experiences on roles of dairy boards in the region that may be relevant as benchmarks in facilitating dairy knowledge and information systems. ^1^ The authors could perhaps go further to look at performance of dairy boards in other LMICs in this respect (e.g., India?). The experiences might help the authors to re-evaluate the risk associated with the key recommendation of (re-) establishing a dairy board in Ethiopia to play similar roles. I guess the report is available from FAO’s repository.

- Is the study design appropriate and does the work have academic merit?

Yes, on both counts. The article describes a participatory process for assessing data needs that is appropriate for the objective of strengthening sectoral capacity within a country. It has academic merit in terms of describing a process relevant elsewhere, providing a novel focus on an information sciences issue relevant to development. It could be strengthened from an academic perspective in terms of (1) establishing its fit in information sciences; (2) evaluation of strengths and weaknesses – as noted in the methods section, but not followed up in the results or discussion sections – or lessons learned during the study, beyond the results achieved specific to the Ethiopian context; and (3) displaying more fully the results consistent with the study objective, especially with respect to relevance of different types of data to the various stakeholders.

- Are sufficient details of methods and analysis provided to allow replication by others?

Yes, the method in terms of process is well described, including the development of the data collection instrument in the form of an interview check-list. The study basically stops at the collection of data and the participatory review and could offer more in terms of how the data can be effectively analysed to inform next steps and what principles to apply to such an analysis.

- If applicable, is the statistical analysis and its interpretation appropriate?

Not applicable.

- Are all the source data underlying the results available to ensure full reproducibility?

Yes, the interview check-list and stakeholder workshop invitation are provided. Could be strengthened by: (1) a more detailed description of how the individual workshop sessions were conducted; and (2) referencing a workshop report that would serve as confirmation and a record of the recommendations agreed by the participants.

*We can provide an anonymised workshop report if required.*

- Are the conclusions drawn adequately supported by the results?

Partly. The conclusions report the general recommendations emerging from the participatory process and are consistent with the account provided by the study authors. It is not clear, though, whether the recommendations represent the explicit recommendations representing the consensus of the workshop participants – which would benefit from reference to a workshop report – or also the recommendations of the study authors: it would be useful to clarify this point. Also, importantly, the discussion and conclusions do not address risks associated with the recommendations especially in terms of repeating previous failures, including ‘who pays, who benefits?’ and potential for un-intended consequences (as have occurred in neighbouring Kenya). In particular, the risk of lack of political will – often a threat – merits highlighting going by the experiences from Tanzania and Kenya. The stakeholder ambition to have a strong autonomous dairy board in Tanzania that could play this role was thwarted at the last minute without further reference to them. Clauses in the dairy bill that envisaged autonomy and a stakeholder driven dairy board were quashed by the government before its enactment, resulting in a body that is essentially a government agency. This is the reason stakeholders started to pursue a different private sector driven approach – the Dairy Development Forum - to fulfil their initial ambitions. More attention should be given to why the proposed ambitious data collection systems would be expected to achieve wider uptake and be sustained.

**Review**

 This study addresses the critical need for data to inform dairy development in Ethiopia by describing a process for assessing those needs and related capacities across the range of relevant actors in the dairy sector. In addition to confirming stakeholder recognition of the need for such data, the process facilitated stakeholder identification of a strategy to begin addressing the needs by establishing a national dairy board that could offer a role in taking responsibility for coordinating the various interests and capacities across the sector, recognizing the risk that it could be captured by government interests. The contribution of the study is therefore to provide a methodology and an example of its application as a participatory process to strengthen sectoral data generation and management that can be replicated for other contexts, whether geographically or for other sectors.

 The study would be further strengthened from addressing four key areas:

- The study approaches the data issue as a development problem, using a review of literature to justify the data issue as a major constraint to dairy development in Ethiopia. It should also recognize that the data issue falls into the domain of information sciences – not necessarily to do an extensive literature review, but to at least acknowledge there is a relevant literature there, too. 
- The objective of the study is “to establish the data needs of dairy sector stakeholders in Ethiopia, and to characterize them by data type, use and relevance to other stakeholders, as a fundamental priority for the sector as it seeks to respond to the rapidly growing demand for dairy products in Ethiopia.” Data presented in the article is limited to a list of data needs and a list of dairy sector stakeholders. These are very useful as baseline information but do not cover the more specific data that appears to have been collected regarding data type, use and relevance to the various stakeholders. A summary of these findings would be expected to be presented as part of the article to serve as a critical starting point for the follow-up analysis and actions recommended by the study. If this information was indeed collected, either Table 1 or Table 2 could be expanded to provide more detail about the type of data (scope or resolution, frequency) and relevance to each stakeholder group.  Also consider elaborating the purpose bullets in the methods section on a) ‘strengths and weaknesses of existing data and data sources’, and b) ‘capacity building needs for better future services’ where it appears the role of the dairy board is envisaged.
- The Results section of the article mentions that “there have been several failed attempts” presumably referring to establishing a national dairy board. One of the reviewers is familiar with such a failed attempt a decade earlier as part of the USAID funded ILRI/ASARECA work to harmonize dairy policies in eastern Africa. Ethiopia was dropped half-way through the process because stakeholders could not hold a national meeting to consider proposals if such a consultation was not driven by government. This suggests that there is a significant risk of history repeating itself if the recommendation to reactivate a national dairy board is followed blindly. It would therefore be important to characterize this risk more fully and consider the factors that could undermine or strengthen the sustainability of both the dairy board and the various data collection efforts proposed. There would be value to some additional discussion of the costs and benefits of the dairy board and the data collection efforts from the perspectives of the different stakeholder groups to qualify what otherwise might be viewed as a high-risk recommendation. An additional reference to cite in this regard is the 2011 FAO report on ‘Dairy Development Institutions in East Africa - Lessons learnt and options’ with experiences on roles of dairy boards in the region that may be relevant as benchmarks in facilitating dairy knowledge and information systems.  The authors could perhaps go further to look at performance of dairy boards in other LMICs in this respect (e.g., India?). The experiences might help the authors to re-evaluate the risk associated with the key recommendation of (re-) establishing a dairy board in Ethiopia to play similar roles.
- It would be useful if to clarify which analyses and recommendations were generated by the participatory process during the workshop and which were provided by the authors themselves.

*Recommendations were those of participants.*

 More specific suggestions:

**Title**

 The authors may want to consider having the participatory process dimension of the study captured more explicitly in the title and objectives.

*We will change the manuscript title to “ **A multi-stakeholder participatory pilot study of the data demands of the future Ethiopian dairy sector**”.*

**Introduction**

 It would be helpful to explain apparent inconsistencies in bullets in the Introduction and later text between:

- *We will change the manuscript to keep “Milk production is predicted to increase from 167 million to 1,490 million litres” and **remove “Combined interventions are predicted to result in a 93% increase in milk production, from 4,132 to 7,967 million litres”***

- *We will change the manuscript to keep “GDP contribution from dairy will increase from Ethiopian Birr (ETB) 1.1 billion to 10.0 billion” and **remove “Increased contribution of cow milk to GDP from ETB 28 billion to 52.9 billion”.***

Would help to clarify what is meant by “Combined interventions” in the sentence quoted in the preceding bullet.

*We will delete this from the manuscript (as above).*

 The initial Purpose statement “By establishing the data needs of stakeholders, priorities and recommendations could be made for how best to develop the dairy sector of Ethiopia” looks to be more about priorities and recommendations for how data can be generated and managed that would best support dairy sector development rather than directly deciding the development strategy.

*We will change the manuscript to “ **By establishing the data needs of stakeholders, priorities and recommendations could be made for how data can be generated and managed that would best support dairy sector development**”.*

 Constraints from Yilma *et al *2011 are cited in one sentence, then the following sentence cites the FAO (Yilma *et al* 2011) study as if it is a new reference being introduced. It would make sense to consolidate the information from the FAO study in one sentence.

*At the end of the first sentence (which cites Yilma et al.), we will add “ **These authors concluded that the major challenges were poor infrastructure network, inadequate provision of veterinary services and lack of continuous supply of animal feeds throughout the year** (Yilma et al. 2011).*

**Methods**

 For “Clusters were identified by defining the dairy value chains in Ethiopia”, suggest something like “stakeholder clusters were identified according to key roles undertaken by the various actors with dairy value chains in Ethiopia.”

*We will change the manuscript to “ **Clusters were identified according to key roles undertaken by the various actors with dairy value chains in Ethiopia**”.*

 “Contact details were provided by the stakeholders in each of the clusters” could be interpreted as raising an ethics concern, especially given the subsequent GPDR requirements. Could perhaps add that as an issue to help justify the appropriateness of the ethics approval.

*We agree and will change the manuscript to “ **Contact details were provided by the stakeholders in each of the clusters, complying with data protection legislation and ethical approval**”.*

 “Local authors provided lists of suitable participants, based on their local knowledge.” Consistent with other statements about potential bias, it would be useful to explain whether there was potential for bias to be introduced here.

*We will add to the manuscript “…local authors provided lists of suitable participants, based on their local knowledge. **Whilst** **there was the potential for bias, this was addressed by ensuring the widest range of participants possible**”.*

 Eight clusters listed initially, but workshop refers to five stakeholder groups: would be helpful to note and explain the change.

***For the workshop, the inputs and services stakeholders (feeds, genetics and health) were clustered for organisational purposes, bringing the eight clusters to five.*** *We can add this to the manuscript at the end of the third paragraph of section ‘Stakeholder workshop’.*

 The structure is a bit confusing after “The groups were asked to identify the different data needs, consolidating them under the following headings”: some of the bullets are repeated in the following paragraphs, some are not. Would be useful to clarify the distinction between the bullets and three subsequent paragraphs.

*The bullets provide the overall framework, the ensuing paragraphs add detail to certain bullets.*

 “Synthesis of outcomes: …. Relevant policy makers were consulted on the findings and plans.” Merits brief explanation why policy makers were targeted rather than all stakeholders.

*This was a mistake – we will change the manuscript to “ **All stakeholders were consulted on the findings and plans**”.*

**Results**

 Stakeholder interactions: starts by repeating information relevant to the methods section, which could be reduced. Would benefit from more evaluation of how it was implemented compared to the design/plan, e.g., adaptations, challenges.

*We believe that the initial information merits repeating; this is the results section, and we are reporting synthesised products.*

 Composition data are presented for the pre-workshop interviews; it would be useful to have workshop participant numbers also presented by cluster/stakeholder group.

*Apologies, but this information is no longer available.*

 Data needs: not clear if synthesis was part of methods or impromptu; it would be useful to have more clarity of any changes made in the method and what the results revealed. Alignment of this and subsequent paragraphs with the headings and the bullets in the methods section may also improve clarity

*We will add to the manuscript: “…a synthesis of data needs by the different stakeholder clusters was conducted, and categorised under headings (Table 1). **A methodological approach was taken, which was refined during the workshop process**”.*

 Table 1: It is stated in the text that “The objective of this study was to establish the data needs of dairy sector stakeholders in Ethiopia, and to characterize them by data type, use and relevance to other stakeholders, as a fundamental priority for the sector as it seeks to respond to the rapidly growing demand for dairy products in Ethiopia.” Results are not presented, though, regarding the “relevance to other stakeholders”. Table 1 could be expanded to include this information with extra columns noting which stakeholder group may have expressed the need and which other stakeholder groups find it relevant – as critical information when designing data collection/coordination mechanisms. Or in Table 2, indicated which data categories were considered a priority for each stakeholder.

*We do not have this level of detail; all implications of inter-stakeholder relevance to each other were stated.  If relevance was not brought up, it was not noted.  The objective statement was indeed the target, but certain components were not identified by the groups.*

 If any information was collected on how to overcome weaknesses in the institutions in playing their roles, this should also be reported.

*Within-institutional weakness were not made explicit, for obvious political reasons.*

 Would be useful to cite some examples of “evidence-based decisions” and by which stakeholder.

*We did not record greater detail.  We already state that data were used for production planning, marketing and investment decisions, policy formulation, system harmonisation, allocation of resources to their best use, and improving the production and productivity in the dairy sector.  They were also used for proper monitoring and evaluation by the regulatory system.*

 Data use: “They were also used for proper monitoring and evaluation by the regulatory system” Would be useful to cite a couple of examples of what this might refer to.

*We do not have any recorded details.*

 Data collection: “… and general principles of seeking permission of owners/creators of data to acknowledge data sources in any form of use.” Incomplete, missing something like ‘would apply’ or ‘would need to be respected’.

*We will change the manuscript to “and general principles of seeking permission of owners/creators of data to acknowledge data sources in any form of use **would apply**”.*

 Institutional arrangements Table 2: While the actors are listed, it would be useful to have a sense of the role they seem themselves or others see them playing within the sector (and were these the same?), and possibly clustering them in the table accordingly. Presumably, this type of information would have been collected and validated during the workshop. It would also be useful to report in the table any relevant strengths and weaknesses for the individual institutions identified by the stakeholders.

*This detail was beyond the scope of the study, and also the political sensitivity of publishing individual institutional strengths and weaknesses.*

 There appears to be repetition between the paragraph entitled ***Where a dairy sector database should be housed and managed*** and the second paragraph preceding it: both discuss candidate institutions for housing the database and so should be merged.

*We will merge these paragraphs **, adding the paragraph** “It was suggested by the policy and regulatory stakeholders group that CSA and MoALR…” **to follow on from the** ‘Where a dairy sector database should be housed and managed’.*

 Housing a database: “…it was intended to establish a unit within the sector for livestock resources, to handle the dairy database.” Would be good to clarify who has the intention and if the MoALR, would they establish it within the ministry or just somewhere ‘within the sector’?

*This section already states that the MoALR was suggested, however there was concern that there may be a lack of capacity to develop and manage such datasets.  Despite this concern, it was intended to establish a unit within the sector for livestock resources, to handle the dairy database.*

 “… there have been several failed attempts” Presumably refers to establishing some type of a national dairy board, but would be good to specify for sake of clarity. Also would be useful to clarify who made the attempts.

*The issues regarding previous attempts to establish a dairy board are presented, and some of the associated issues.  We could add “ **such an initiative has also encountered hurdles in other countries of eastern Africa, even in countries with a much higher dairy production per cow (Kurwijila L.R., Bennett A.; Dairy development institutions in East Africa – Lessons learned and options.  Food and Agriculture Organization of the United Nations. 2011).***

 “… a variety of factors have held back the development of the sector, namely strong associations (producers cooperatives) that support development, and challenges in access to land.” Suggests strong associations have held back development; or was it meant to be ‘lack of strong associations'?

*We will change the manuscript to “the **lack** of strong associations that promote development, and the differing views on the role of government leadership”.*

**Discussion**

 “Sector-specific needs were identified and should be elevated in detail as they are currently somewhat generic, requiring further shaping within each stakeholder group” Not sure what ‘elevated in detail’ means.

*We believe that we have made and discussed this statement adequately.*

 “… the sector for livestock development under the MoALR” What is a sector under a ministry? Sounds like a unit or equivalent to the other agencies.

*We are unclear on this query.*

 “… a newly established National Dairy Board” implies it already exists. Should it be: ‘proposed’?

*Agreed, we will change the manuscript to “and a **proposed** newly established Dairy Board”.*

 “Further data analyses and modelling could be of value in supporting evidence-based decision making for dairy sector development and will be explored by Livestock Data for Decisions (LD4D) data scientists.” Would be useful to clarify how this fits with the focus on creating sustainable domestic institutional capacity – should it be more explicitly framed as a one-off strategic technical support or input for designing?

*We would agree and will change the manuscript, in the conclusion, to say **the value of seeking one-off strategic technical support on design options**.*

 Some of the workshop recommendations might be interpreted as ‘wishlist’ expressions of ‘want’ as sometimes come out of such workshops. The Discussion is meant to be more analytical, so is it possible to help the reader distinguish?

*Part of the process was for stakeholder groups to provide specific relevant suggestions and ideas, but the value of “thinking out of the box” was welcomed.*

 Focus in the Discussion is on establishing a new entity, but with little attention or analysis of its potential to be sustained. It would benefit from more discussion around the information collected/results and action needed that would support addressing sustainability issues during the design, especially given the comment made about previous failed attempts.

*We agree and will add to the conclusion “ **The long-term sustainability of any new institutional arrangements need to be considered in addition to the functionality and business approach advocated by many of the stakeholders**”.*

**Conclusion**

 “… reactivation of a national dairy board was strongly endorsed.” This implies one had existed; it would be useful to clarify whether one previously established that was being reactivated, or whether it was the proposal for one that was being reactivated.

*The old Board was during a previous era, under Haile Selassie.  We could **remove the word ‘reactivation’**.*

 While the recommendation to re-establish a national dairy board is ‘likely to be seen as acceptable in the current political climate’ as noted by the authors, it would be useful to consider a recommendation that would mitigate the risks of lack of political will and government capture and overcome previous failures to establish such a body. This might entail some political economy analysis and recommended engagement with key actors based on the findings.

*We agree and will add “ **However, it would be useful to mitigate the risks of lack of political will and government capture, and overcome previous failures in the establishment of a National Dairy Board; this would likely entail some political economy analysis and recommended engagement with key actors based on the findings**”.*

 Again, the Conclusion seems to assume ‘build it and they will come’, i.e. assuming roll-out will automatically ensure uptake and sustainability. It would be useful to flag any risks and highlight what is needed to avoid repeating earlier failed attempts.

*Agreed, we will add “ **It would be worth conducting a SWOT analysis on the establishment of a Dairy Board, clearly identifying the strengths, weaknesses, opportunities and threats, and engaging multiple stakeholders in this process**”.*
